# Bortezomib Plus Melphalan-Induced Cardiomyopathy Presenting as Sinus Tachycardia and Systolic Heart Failure

**DOI:** 10.7759/cureus.9488

**Published:** 2020-07-31

**Authors:** Parth J Sampat, Sana Riaz, Fidel Martinez, Dana Aiello

**Affiliations:** 1 Internal Medicine, State University of New York Upstate Medical University, Syracuse, USA; 2 Cardiology, State University of New York Upstate Medical University, Syracuse, USA

**Keywords:** cardiomyopathy, bortezomib, melphalan, sinus tachycardia, cardiotoxicity, systolic heart failure

## Abstract

Chemotherapy-induced cardiotoxicity is a known condition, however, bortezomib and melphalan do not typically cause cardiotoxicity. With the rise in the use of newer chemotherapeutic agents, it is important to identify and understand the cardiac implications of chemotherapeutic agents. We present a case of a 70-year-old female with no known significant cardiac history presenting with partially reversible cardiomyopathy with initial presentation only being as sinus tachycardia.

## Introduction

Multiple myeloma (MM) is a neoplastic proliferation of plasma cells [1]. The use of autologous stem cell transplantation and the availability of treatments such as immunomodulatory drugs and proteasome inhibitors have led to an increase in survival times [1]. Chemotherapy has been associated with cardiotoxicity in a variety of manners, which include hypertension, arrhythmias, heart failure, and so on [2]. Anthracyclines are widely considered as cardiotoxic which has limited its use [3]. Newer chemotherapy agents have lesser cardiovascular adverse events. Bortezomib is approved for the treatment of multiple myeloma and mantle cell lymphoma and is considered safer in terms of cardiovascular profile. Reports are suggesting that bortezomib is associated with cardiomyopathy and cardiotoxicity, however, is still considered rare [4-9]. The incidence of cardiotoxicity with the use of bortezomib-based regimens has been between 0-17.9% [7]. Melphalan is used for conditioning prior to hematopoietic stem cell transplant for the treatment of multiple myeloma [10]. The use of bortezomib and melphalan together has been found superior for the treatment of multiple myeloma [11]. Cardiomyopathy has been reported with high dose melphalan conditioning for autologous stem cell transplant with the incidence being approximately 1.6% with the use of melphalan for MM [12]. In biological experiments on rats, it has been found that bortezomib at higher concentrations increases the cytotoxic effects of melphalan on cardiac myocytes [13]. As the use of these medications has been increased in the past few years, understanding rare adverse effects are important, particularly cardiovascular toxicities. We present a case of multiple myeloma developing cardiotoxicity following the use of bortezomib and melphalan.

## Case presentation

A 70-year-old female with a diagnosis stage II multiple myeloma (MM) by international staging system for multiple myeloma, high risk according to mSMART (Mayo stratification of myeloma and risk-adapted therapy) classification, on chemotherapy with lenalidomide, bortezomib, and dexamethasone presented to our office for a pre-stem cell transplant appointment. In addition to multiple myeloma, she had a past medical history of hyperlipidemia and anxiety. She denied cigarette or alcohol use. She did not have any known cardiovascular history. Family history was significant for myocardial infarction in a brother at the age of 35 and myocardial infarction in her mother at the age of 50. A routine echocardiogram obtained one month prior to stem cell transplant revealed a normal left ventricular ejection fraction of 62% with a resting tachycardia (Video 1). Two months before she began chemotherapy, her heart rate was 77 bpm on electronic medical records (EMR). She received three cycles of chemotherapy before she presented to the office.

**Video 1:** Four chamber view showing ejection fraction of 62% and resting tachycardia

**Figure 1:**
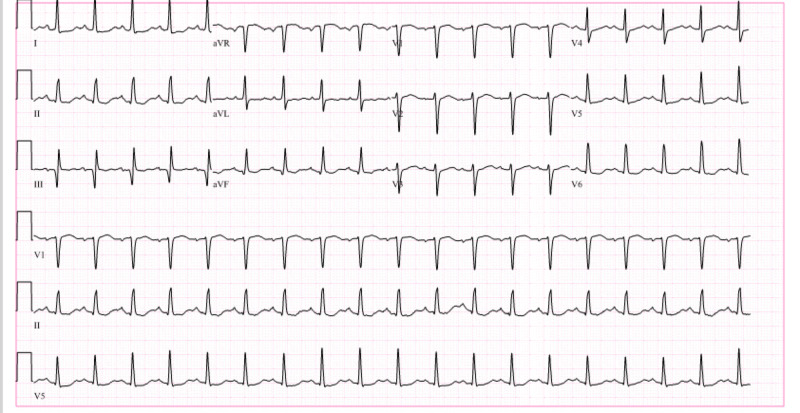
Sinus tachycardia with heart rate of 113 beats per minute and non-specific ST-T wave abnormalities

She was admitted to the hospital for an autologous stem cell transplant. Her vitals were significant for a heart rate (HR) of 124 bpm, temperature (T) 37 C, respiratory rate (R) 16/min, and blood pressure (BP) 120/69 mmHg. Baseline electrocardiogram (EKG) at the time of admission showed sinus tachycardia with a heart rate of 113 bpm without any ST-T abnormalities (Figure 1). Her physical examination was significant for tachycardia, regular rhythm, and a grade II/VI systolic murmur in the mitral region. The respiratory examination was clear to auscultation bilaterally. No peripheral edema or jugular venous distension was noted.

She was conditioned with melphalan, followed by a successful autologous stem cell transplant. She remained persistently tachycardic despite IV fluids. Sixteen days after stem cell transplant, bibasilar crackles were found via physical exam. Peripheral edema and jugular venous distension were noted. She did not have orthopnea, paroxysmal nocturnal dyspnea, or shortness of breath on exertion.

**Figure 2:**
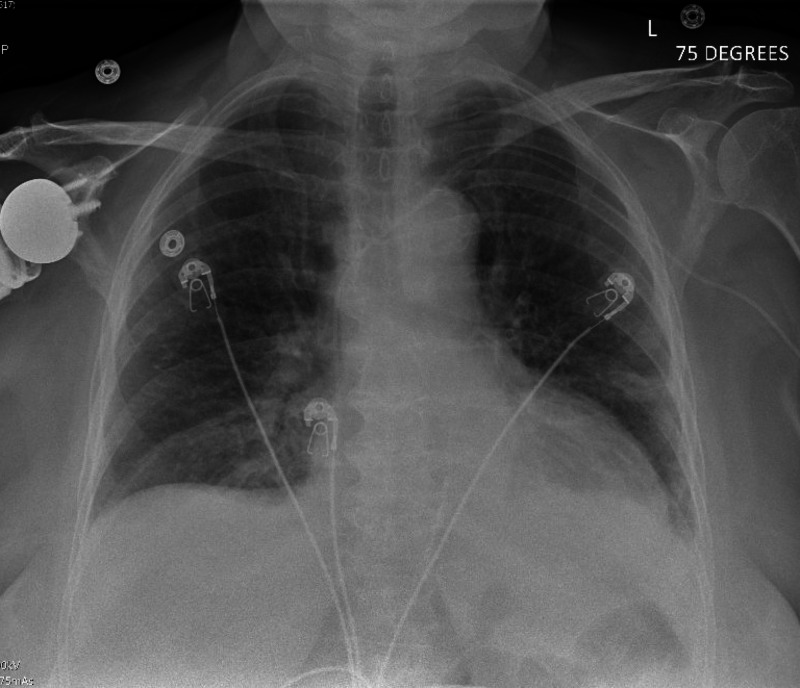
Chest X-ray showing pulmonary vascular congestion and bilateral small pleural effusions

**Figure 3:**
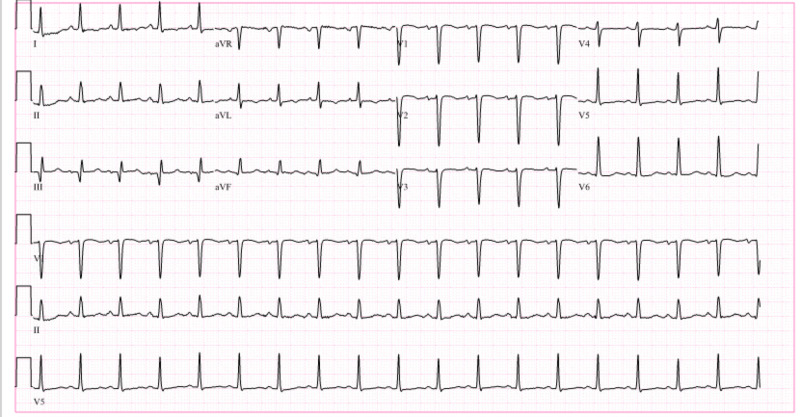
Electrocardiogram (EKG) showing sinus tachycardia with heart rate of 109 beats per minute

N-terminal pro b-type natriuretic peptide (NT-proBNP) obtained at the time was elevated at 12,436 pg/ml. A chest X-ray showed pulmonary edema and small bilateral pleural effusions (Figure 2). Electrocardiogram revealed sinus tachycardia with HR of 109 bpm and no evidence of ischemia (Figure 3). A repeat echocardiogram showed worsening systolic function with a calculated ejection fraction of 24.2% and diffuse hypokinesis of all walls (Video 2). Angiography was not obtained due to suspicion of chemotherapy-induced cardiomyopathy as she had global hypokinesis without segmental wall motion abnormalities.

**Video 2:** Four chamber view showing calculated ejection fraction of 24.2% and diffuse hypokinesis of all walls

The patient was initiated on metoprolol tartrate 25 mg twice a day, ivabradine 5 mg twice a day, and losartan 25 mg daily to control tachycardia and medical management of systolic heart failure with reduced ejection fraction. IV furosemide was given for pulmonary congestion while hospitalized. She was discharged on metoprolol succinate 200 mg daily, ivabradine 5 mg twice a day, losartan 25 mg daily, and furosemide 20 mg daily.

Two months later, during her follow-up visits, her heart rate remained between 70-80 bpm. A repeat echocardiogram performed four weeks later showed partial resolution of heart failure with a visually estimated ejection fraction of 45-50%.

## Discussion

Heart failure in this patient is believed to be due to bortezomib and exacerbated by melphalan. Sinus tachycardia and heart failure were attributed to the use of these agents since she had no sinus tachycardia before starting chemotherapy. This case demonstrates the rare adverse effects of bortezomib and melphalan when used concurrently.

Bortezomib is a dipeptidyl boronic acid and has a role in worsening ischemic heart disease [14, 15]. It is a potent and reversible inhibitor of 26S proteasome. Reduced proteasome activity causes increased apoptosis in smooth muscle cells, which can result in plaque instability and can lead to an increased likelihood of plaque rupture and ischemic complications [7]. Furthermore, biological experiments have shown that bortezomib can cause mitochondrial abnormalities in the cardiac myocytes leading to a decrease in adenosine triphosphate (ATP) synthesis and reduced contractility [13]. Thereby, explaining the underlying etiology of heart failure with the use of bortezomib. However, the effect of bortezomib on cardiac myocytes is reversible since there is no associated apoptosis.

Melphalan cardiotoxicity presents more commonly as atrial fibrillation and supraventricular tachycardia [16], but ventricular arrhythmias have also been reported [17]. There is also an association between melphalan and heart failure [12, 18, 19], but the underlying mechanism is unknown. A cumulative dose of bortezomib is an important consideration because it has been associated with an increased risk of cardiac side effects [7]. Moreover, in experiments with rats, the concurrent use of melphalan along with higher concentrations of bortezomib has been found to increase the cytotoxic effects of melphalan on cardiac myocytes [13]. Our case thus demonstrates the increased risk of heart failure and other cardiac toxicities with the concomitant use of bortezomib and melphalan.

## Conclusions

Cardiomyopathy in our patient was likely a result of the concurrent use of bortezomib and melphalan. Improvement of ejection fraction within four weeks of completion of treatment and starting medical management of heart failure shows reversibility. Sinus tachycardia, with the commencement of bortezomib, has not been observed in prior studies. We believe that even subtle signs such as sinus tachycardia should be given importance for early identification of cardiotoxicity. Thus, we recommend close monitoring of cardiac parameters in patients treated with bortezomib and melphalan.
